# Engineering His-Tagged Senecavirus A for One-Step Purification of Viral Antigens

**DOI:** 10.3390/vaccines10020170

**Published:** 2022-01-22

**Authors:** Junhao Fan, Peiyu Xiao, Dongni Kong, Xinran Liu, Liang Meng, Tongqing An, Xuehui Cai, Haiwei Wang, Li Yu

**Affiliations:** 1 State Key Laboratory of Veterinary Biotechnology, Harbin Veterinary Research Institute, Chinese Academy of Agricultural Sciences, Harbin 150069, China; ffanjh@163.com (J.F.); jiachujiepou@163.com (P.X.); 13035553899@163.com (L.M.); antongqing@caas.cn (T.A.); caixuehui@caas.cn (X.C.); 2 China Institute of Veterinary Drug Control, No. 8 Nandajie, Zhongguancun, Haidian, Beijing 100081, China; kongdongni@163.com; 3 Regeneron Pharmaceuticals Inc., 777 Old Saw Mill River Road, Tarrytown, New York, NY 10591, USA; xinranliu603@gmail.com

**Keywords:** Senecavirus A, His-tagged virus, antigen purification

## Abstract

Senecavirus A (SVA) is a picornavirus that causes vesicular disease in swine, and the inactivated vaccine is used to prevent and control SVA infection. To develop a new chromatography strategy for the purification and concentration of SVA vaccine antigens, we inserted a 6×His-tag at the VP1 C-terminal of the SVA/HLJ/CHA/2016 in an infectious clone to rescue a His-tagged SVA. The constructed and rescued recombinant virus, named as rSVA-His, exhibited similar growth kinetics to that of its parental virus. In addition, the expression of a 6×His-tag on the surface of SVA showed genetic stability in cell passages in vitro, which allowed one-step purification of SVA antigens by Ni^2+^ affinity columns. Furthermore, the immunogenicity of the inactivated rSVA-His was evaluated by inoculating rabbits and detecting neutralizing antibodies. The animals receiving two doses of the inactivated rSVA-His emulsified with oil adjuvant developed a high titer of neutralizing antibodies, indicating that SVA VP1 is tolerant to His-tag insertion without detriment to its antigenicity. In summary, the constructed 6×His-tagged SVA may offer a feasible approach to the affinity purification and concentration of antigens in the process of SVA inactivated vaccine production.

## 1. Introduction

Senecavirus A (SVA), formerly named Seneca Valley Virus (SVV), is one of the causative agents of vesicular diseases in swine. Outbreaks of SVA infection have been consistently reported in some parts of the world, including Asia, and thus poses a significant threat to the pig industry [1]. SVA is the single member of the genus Senecavirus within the family Picornaviridae and shares some common features with other picornaviruses: (1) a non-enveloped virus; (2) a single-stranded positive-sense RNA genome flanked by an internal ribosomal entry site at the 5′ end and a polyadenylated tail at the 3′ end; (3) a large, single open reading frame encoding a polyprotein; (4) a translated polyprotein precursor with the standard L-4-3-4 layout being processed into mature proteins by virus-encoded proteinases [2]. These mature proteins include four structural proteins (VP1-–VP4) and eight nonstructural proteins (L, 2A-2B-2C-3A-3B-3C-3D) [2]. VP1, VP2, and VP3 are on the external surface of the virus capsid of SVA, while VP4 lies on its inner surface [3].

SVA was first isolated from a cell culture contaminant in 2002 [2]. Studies have suggested that SVA infection is associated with the sporadic outbreaks of vesicular diseases with unknown etiology, called porcine idiopathic vesicular disease (PIVD) in the early days [4,5]. Subsequently, positive cases of SVA were detected in Brazil in late 2014 and 2015 [6,7]. In the summer of 2015, several SVA infections in pigs were reported in the United States that resembled those seen in the outbreaks in Brazil [8,9]. With cases appearing in the United States, China, Canada, Thailand, Colombia, and Vietnam, SVA might circulate globally [10,11,12,13,14,15]. The symptoms observed in SVA infection are vesicles on the snout and/or feet of affected animals, which are clinically indistinguishable from other high-consequence vesicular diseases of swine, including foot and mouth disease (FMD), swine vesicular disease (SVD), vesicular stomatitis (VS), and vesicular exanthema of swine (VES) [16]. During the acute phase of SVA outbreaks on farms, the incidence of vesicular lesions varies by the herd, ranging from 10% to 90% [17]. It affects feed intake and movement once vesicles rupture or erode on the snout, oral mucosa, and feet. It was also reported that SVA is linked to increased mortality in neonatal piglets with high morbidity [18].

The most economical and effective way to prevent infectious diseases is through vaccination. Challenges encountered in developing safe, cost-effective, and marker vaccines that allow for distinguishing infected from vaccinated animals prevent their rapid development. Although there are no commercially available vaccines against SVA infection, recent studies described the progress of SVA vaccine candidate development, including one inactivated vaccine and one live attenuated vaccine at the laboratory scale, and an immune efficacy evaluation of both candidates demonstrated that they provided protection against homologous or heterologous virus challenge [19,20]. A major concern of using live attenuated vaccines is that they may cause disease due to evolutionary reversion, although stronger immune responses are usually elicited. For this reason, inactivated vaccines are considered safer and more stable. To date, the majority of commercially manufactured animal vaccines against viral pathogens are inactivated whole-cell vaccines [21]. Since inactivated whole-cell vaccines are usually expressed in a complex biological matrix with a wide range of impurities such as cell debris, host-cell-derived contaminants (e.g., proteins, DNA, and endotoxins) as well as viral non-structural proteins, causing difficulties in distinguishing infected from vaccinated animals, downstream processing must comply with strict purity requirements [22]. Additionally, relatively simple purification steps should enormously benefit the vaccine product development process as process scale-up, manufacturing cost, and consistent product quality can be better controlled [23]. A highly desirable purification technology with the potential to meet high-purity criteria in a single step is immobilized metal affinity chromatography (IMAC), which is based on the interaction between an immobilized metal ion (Cu^2+^, Zn^2+^, Ni^2+^, Fe^3+^, or Co^2+^) and electron donor groups located on the surface of proteins. IMAC has been widely developed for the purification of vaccines and gene therapy vectors, such as foot and mouth disease virus (FMDV), adeno-associated virus (AAV), herpes simplex virus type 1 (HSV-1), and murine leukemia virus (MuLV) [24,25,26,27].

In this study, we designed, produced, and characterized a recombinant SVA inserted with a 6×His-tag for one-step purification of SVA antigens via gravity nickel nitrilotriacetic acid (Ni-NTA) column chromatography. Our results showed that the His-tagged SVA is genetically stable and exhibits similar growth kinetics to its parental virus. Importantly, the insertion of the His-tag in the VP1 did not affect the immunogenicity of SVA. Thus, the data presented here provide a proof of concept for an effective vaccine strain against SVA infection with high purity in a single step to potentially accelerate the vaccine product development process.

## 2. Materials and Methods

### 2.1. Ethics Statement

This study was carried out in strict accordance with the recommendations in the Guide for the Care and Use of Laboratory Animals of the Ministry of Science and Technology of the People’s Republic of China. All animal experiments were approved by the Animal Care and Ethics Committees of Harbin Veterinary Research Institute (HVRI), Chinese Academy of Agricultural Sciences (CAAS). Protocols for the animal study were approved by the Committee on the Ethics of Animal Experiment of the HVRI, CAAS (protocol number 201104-02).

### 2.2. Cell Lines and Viruses

Baby hamster kidney strain 21 cell line (BHK-21) was maintained in Dulbecco’s modified Eagle’s medium (DMEM; Gibco Grand Island, NY, USA) supplemented with 10% fetal bovine serum (FBS; HyClone Laboratories, Inc., South Logan, UT, USA) at 37 °C in 5% CO_2_ in a humidified atmosphere. The wild-type SVA/HLJ/CHA/2016 (GenBank accession number: KY419132) was obtained from finishing pigs on a farm of Heilongjiang Province in northeast China in 2016, as described previously [11]. The high-fidelity variant SVA-I212V/S460L cDNA clone was used as the backbone to construct His-tagged recombinant virus [28].

### 2.3. Construction and Recovery of rSVA-His Recombinant Virus

The 6×His-tag was inserted at the C-terminal of VP1 by overlap PCR using the SVA-I212V/S460L backbone, which is the high-fidelity variant with the double amino acid changes I212V and S460L in its RNA-dependent RNA polymerase (3Dpol) region. The primers used in this study are listed in Table 1. This full-length cDNA clone was designated as pSVA-His. BHK-21 cells seeded in a 6-well plate were transfected with pSVA-His using Lipofectamine 3000 reagent (Life Technologies, New York, NY, USA), following the manufacturer’s instructions. The cytopathic effect (CPE) was monitored daily after infection. Recombinant viruses were harvested when a significant CPE was observed. The recovered viruses were serially passaged ten times in BHK-21 cells, and the stability of the introduced 6×His-tag was confirmed by sequencing of the VP1-2A region.

### 2.4. Replication Kinetics of Recombinant Virus

To determine viral replication kinetics, viral growth kinetics were assayed in tissue culture models of infection. BHK-21 cells were seeded in a 6-well plate until cell monolayers reached 80~90% confluency and were then infected with either wild-type virus or recombinant virus at a multiplicity of infection (MOI) of 0.01. After incubated for 1 h at 37 °C, the plates were washed with phosphate-buffered saline (PBS) three times and then cultured by DMEM with 2% FBS. The plates were harvested at different times. Subsequently, the viral titer was determined via a 50% tissue culture infective dose (TCID_50_) assay, which values were calculated by the Reed–Muench formula.

### 2.5. IMAC Purification of Inactivated rSVA-His Particles

Before purification, 20 mL supernatant of His-tagged virus cultured by BHK-21 cell was inactivated by binary ethylenimine (BEI; Sigma, St. Louis, MO, USA). A column filled with Ni-NTA agarose (Genscript, Jinsirui Biotechnology Co., Ltd., Nanjing, China) was used to purify the inactivated rSVA-His recombinant virus. The virus sample was centrifuged at 6000× *g* for 30 min to remove excess cell debris prior to loading onto the column equilibrated with lysis equilibrate buffer (50 mM NaH_2_PO_4_, 300 mM NaCl, pH 8.0). The column was then washed with 8 column volumes of wash buffer (50 mM NaH_2_PO_4_, 300 mM NaCl, 10 mM imidazole, pH 8.0). Fractions of wash buffer after flow-through were collected and assayed for further purity analysis. Then, the bound virus was eluted with a linear gradient (5 column volumes) containing 100 mM to 500 mM imidazole (i.e., E1 = 100 mM imidazole, E2 = 200 mM imidazole, E3 = 300 mM imidazole, and E4 = 500 mM imidazole in 50 mM NaH_2_PO_4_, 300 mM NaCl, pH 8.0). The purified virus was negatively stained. Transmission electron microscopy images were collected with a Hitachi H7650 transmission electron microscope (Hitachi, Ltd., Tokyo, Japan).

### 2.6. Indirect Immunofluorescence Assay (IFA)

IFA was performed as described previously [28]. Fixed cells were treated with anti-SVA VP2-specific monoclonal antibody (mAb) 2F5 (1:1000), anti-His mAb, or rabbit serum from the animals immunized with purified protein fractions. After washing with PBS, fluorescein isothiocyanate (FITC)-conjugated goat anti-mouse IgG (Sigma, St. Louis, MO, USA) or Alexa Fluor 488-labeled goat anti-rabbit IgG (Beyotime, Biotechnology, Nanjing, China) was added and incubated for 50 min at room temperature. Plates were washed three times with PBS and examined under an EVOS FL Auto 2 Cell Image System.

### 2.7. SDS-PAGE and Western Blot

Cell lysates or protein fractions purified using metal affinity reagents were loaded and separated under denaturing conditions in a 12% SDS-PAGE gel visualized with Coomassie Blue R-250. For the Western blot assay, proteins were transferred onto polyvinylidene difluoride (PVDF) membranes. The blots were blocked with 5% skim milk in PBS at room temperature, followed by an additional incubation of 1 h with anti-SVA VP2-specific mouse monoclonal antibody, anti-His mAb, or rabbit serum from the animals immunized with purified protein fractions. The antibody dilutions were made in PBS and 1% skim milk. The blots were subsequently washed three times with PBS-T and incubated with secondary IRDye^®^ 800 CW goat anti-mouse IgG or IRDye^®^ 800 CW goat anti-rabbit IgG antibodies, and signal detection was performed using a near-infrared fluorescence scanning imaging system (Licor Odyssey, Lincoln, NE, USA).

### 2.8. Animal Experiments

Nine female New Zealand White rabbits were divided into three groups with three rabbits in each group. In group A, each rabbit was immunized with 10 μg of inactivated SVA, which was purified by sucrose density gradient centrifugation and mixed with oil adjuvant in a total volume of 200 μL. Each rabbit in group B was immunized with 10 μg of inactivated purified rSVA-His by IMAC, with oil adjuvant, and the same volume of DMEM was inoculated as a negative control in group C. A boost injection was given two weeks after the initial prime. Blood was collected at day 0 pre-vaccination and 1, 2, and 4 weeks post-vaccination. Sera were separated after the collected blood was centrifuged at 8000 rpm for 5 min and were stored at −80 °C until further analysis.

### 2.9. Virus Neutralization Test

The neutralizing antibody responses of immune rabbit sera elicited by rSVA-His were assessed using a virus neutralization assay. Specifically, the sera were heat-inactivated at 56 °C for 30 min, and then each 2-fold-diluted serum was incubated with 200 TCID_50_ of SVA-WT for 1 h at 37 °C. Subsequently, BHK-21 cells were added to each well, and plates were cultured for three days. Neutralizing antibody titers were measured by examination of the cytopathic effect (CPE) of the cells and are expressed as log2 (reciprocal of the highest serum dilution capable of completely inhibited SVA-induced CPE). All assays were performed in triplicate and included positive and negative controls in all tests.

### 2.10. Statistical Analysis

The results are expressed as the mean ± standard deviations (SDs). Statistical analysis and data plotting were performed with GraphPad Prism 7.0 software (GraphPad Software Inc., La Jolla, CA, USA) using Student’s *t*-test or one-way ANOVA. *p*-values < 0.05 were considered to indicate statistically significant differences.

## 3. Results

### 3.1. Generation and Characterization of His-Tagged SVA

**Figure 1:**
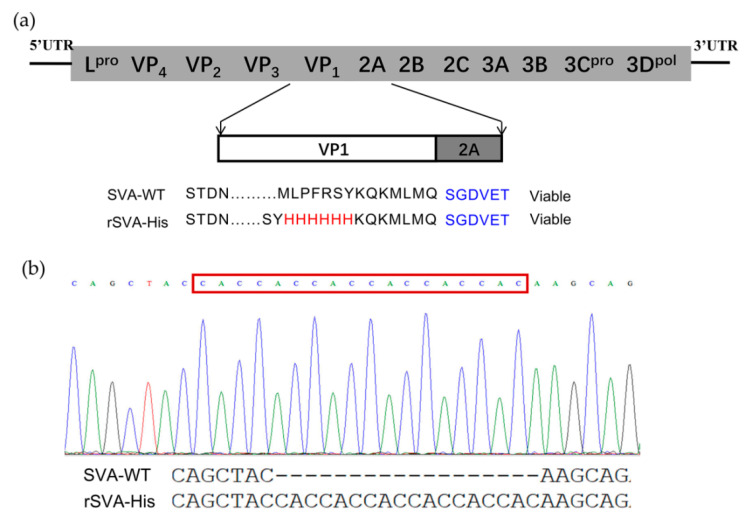
Schematic representation of full-length infectious clone ofrSVA-His and genetic stability analysis. (**a**) The inserted 6×His-tag sequence is shown in red text surrounded by VP1 residues. 2A residues of SVA are shown in blue text. (**b**) Sequence analysis of rSVA-His after ten passages in BHK-21 cells. The nucleotide sequence encoding 6×His-tag (CACCACCACCACCACCAC) is shown.

**Figure 2:**
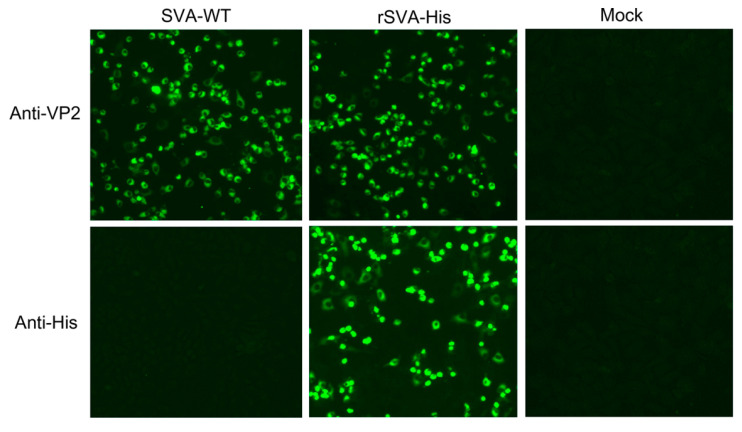
Detection of rSVA-His on immunofluorescence. The BHK-21 cells infected with rSVA-His were incubated with anti-VP2 mAb or anti-His mAb, and detected by the anti-mouse FITC. The BHK-21 cells infected with SVA/HLJ/CHA/2016 were used as the positive controls and normal BHK-21 cells were used as the negative control.

**Figure 3:**
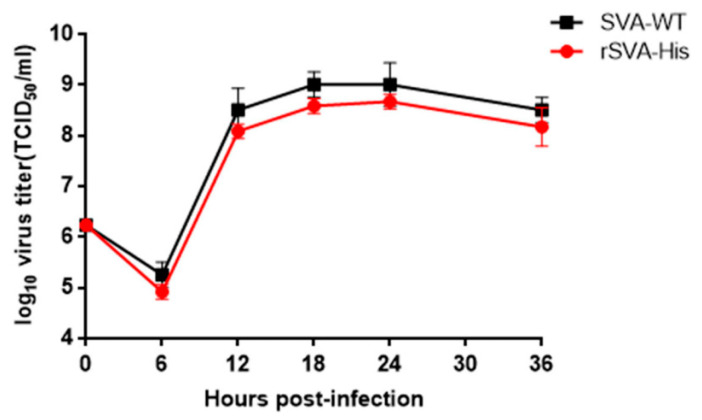
Growth curve of the rSVA-His in BHK-21 cell. BHK-21 cells were infected with rSVA-His and its parental virus. Samples were harvested at different time points post-infection and the infectivity titers were detected. Each data point represents the mean (±SD) of three wells.

To construct His-Tagged SVA, a recombinant SVA mutant that encodes a high-fidelity viral polymerase with I212V and S460L mutations, rSVA-I212V/S460L, constructed by our laboratory previously, was used as skeleton virus to improve the genetic stability of the recombinant virus and the consistency of foreign genes’ expression. A 6×His-tag was inserted in an infectious cDNA clone of rSVA-I212V/S460L by in-fusion PCR with restriction enzyme digestion at the C-terminus of VP1 named pSVA-His, as shown in Figure 1a. The full-length cDNA clone pSVA-His was transfected into BHK-21 cells to rescue the virus. CPEs were observed 48 h after transfection, and the viral supernatant collected 48 h post-transfection was then passaged serially in BHK-21 cells ten times. RT-PCR and sequencing analysis showed that the 6×His-tag was stably inherited after ten serial passages (Figure 1b). The presence of His-tag in the recombinant virus was examined in IFA. The result showed that the rSVA-His in the infected cells could be detected by both anti-His mAb and anti-SVA VP2 mAb, while the anti-His mAb did not react to the SVA-WT in the infected cells (Figure 2). Furthermore, the one-step growth curve demonstrated that the rSVA-His exhibited similar replication kinetics to SVA-WT (Figure 3). These results indicated that the insertion of His-tag did not alter the growth properties of rSVA-His relative to its parental virus, and that the expression of His-tag in rSVA-His replication was maintained stably.

### 3.2. One-Step Purification of the Recombinant Virus rSVA-His Using IMAC

**Figure 4:**
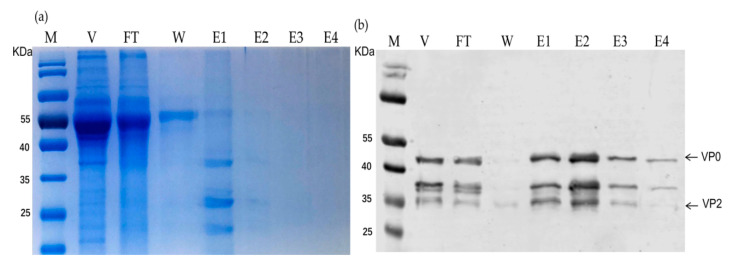
SDS-PAGE and Western blot (WB) analysis of the purified rSVA-His. (**a**) Coomassie Blue-staining of SDS gel to detect the purity of rSVA-His purified in a Ni-NTA column. (**b**) Fractions eluted from the column were analyzed by WB using a monoclonal antibody against SVA capsid protein. Lanes: M, marker; V, crude lysates; FT, flow-through fraction; W, washing fraction; E1 to E4, elution fractions.

We next purified inactivated rSVA-His from culture supernatant in a Ni-NTA column. The viral purity was analyzed by SDS-PAGE followed by Coomassie Brilliant Blue staining (Figure 4a). Most of the contaminated proteins (either cellular debris or serum proteins) was removed from the flow-through step and washing steps without binding to the Ni-NTA column. Only the rSVA-His viral particles bound to the Ni-NTA matrix, which were subsequently eluted with elution buffer containing imidazole in different concentrations. The result indicated that the impurities in the eluted fractions were negligible compared to the impurities in the crude lysate. Furthermore, the presence of SVA particles was confirmed by Western blot using VP2 monoclonal antibody. The results showed that the virus particles purified by IMAC showed a strong VP2 protein signal and that the fraction with the highest yield of SVA particles was eluted at 100–200 mM imidazole (Figure 4b). These results indicated that the 6×His-tag on the capsid protein VP2 of SVA specifically binds to the Ni-NTA column, and this feature facilitates the rapid purification of SVA antigens.

### 3.3. Electron Microscopy Detection of rSVA-His

**Figure 5:**
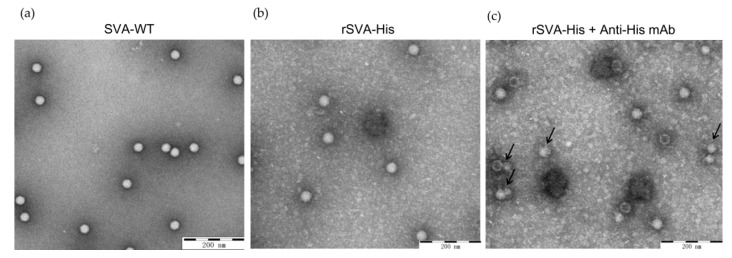
Observation of rSVA-His on transmission electron microscopy. (**a**,**b**) Morphology of rSVA-His and parental virus. (**c**) EM image of rSVA-His incubated with anti-His mAb. Black arrows indicate specific binding of anti-His mAb to rSVA-His.

IMAC may not work if the expressed 6×His-tag is buried within the 3D structure of recombinant SVA during the assembly of viral particles. To verify that the His-tags are exposed on the surface of the virion and thus can be accessible to Ni^2+^, purified rSVA-His was incubated with anti-His mAb for observation on electron microscopy (EM), and SVA-WT was used as control. Both rSVA-His and its parental virus SVA-WT showed a smooth capsid surface in the absence of anti-His mAb (Figure 5). In contrast, the rSVA-His particles exhibited crude capsid surface in the presence of anti-His mAb (Figure 5c), suggesting there is a specific binding of the anti-His mAb to the 6×His-tags expressed by rSVA-His. This result provided evidence for the feasibility of purifying His-tagged SVA from infected cell culture in a single step using the IMAC method.

### 3.4. Evaluation of rSVA-His for Immunogenicity in Rabbit

**Figure 6:**
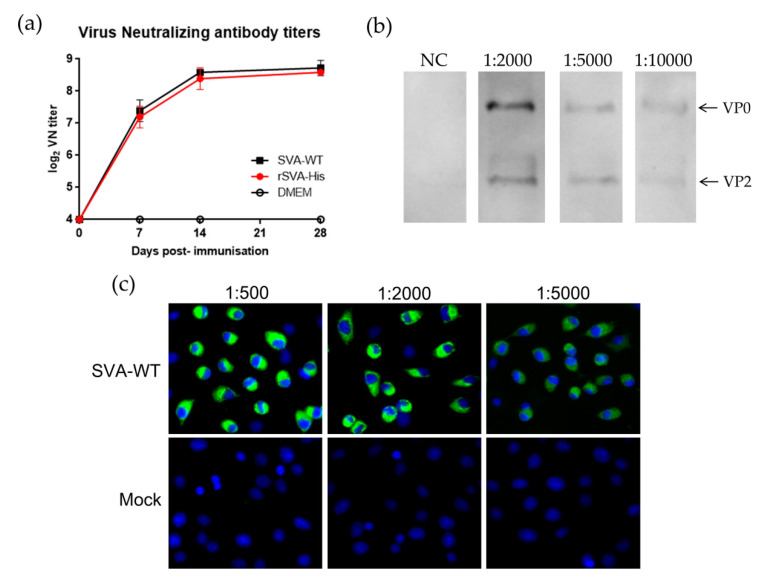
Antibody response of rabbits inoculated with purified rSVA-His. Nine rabbits were randomly divided into three groups (n = 3/group) and intramuscular injection twice with 10 μg purified parental SVA and purified rSVA-His. DMEM was inoculated as a negative control. Blood samples were collected on day 0 pre-vaccination and 1, 2, and 4 weeks post-vaccination. (**a**) Titers of neutralizing antibodies were detected by virus neutralization test and are expressed as a log2 value. (**b**) WB analysis of SVA-WT-infected BHK-21 cells with immunized rabbit serum. The rabbit serum was diluted 2000, 5000, and 10,000 times. Normal cell lysates were used as the control. (**c**) IFA of SVA-WT-infected cells with immunized rabbit serum. SVA-WT-infected BHK-21 cells were incubated with several dilutions (1:500, 1:2000, and 1:5000) of the rabbit serum. Normal BHK-21 cells were used as the control.

In order to examine the effect of 6×His-tag insertion on the immunogenicity of rSVA-His, three rabbits were immunized with purified and inactivated rSVA-His, and its parental virus SVA-WT was used as a control for inoculation. Serum was collected on day 0 pre-vaccination and at 7, 14, 21, and 28 days post-immunization (d.p.i.) for detection of viral neutralizing antibodies. The titers of neutralizing antibodies against the rSVA-His exhibited an average level of 1:384 (8.58 log 2), which is comparable to those against the parental virus SVA-WT, indicating that the rSVA-His retained its immunogenicity in vivo (Figure 6a). More importantly, our laboratory observed that SVA-WT induces higher neutralizing antibody titers in pigs than in rabbits (data not shown). Since rSVA-His and the parental virus share similar structural characteristics and immunogenicity in rabbits, we expected that the His-tagged SVA would provide stronger protection in pigs by inducing potent neutralizing antibodies as significantly as its parental virus. To evaluate the specificity and sensitivity of neutralizing antibodies produced in the immunized rabbit, we analyzed rSVA-WT-infected cell samples using the serum as the primary detection antibody on WB and IFA, separately. The serum of the immunized rabbit diluted 5000- and 10,000-fold still captured SVA antigens in the infected cell culture in IFA and WB (Figure 6b,c). These results suggested that rSVA-His retained the antigenicity of the parental virus and induced similar levels of neutralizing antibodies in the immunized animals.

## 4. Discussion

Since the first outbreak in Guandong province in China in 2015, it has been reported that most of China is affected by SVA infection [29]. SVA infection causes vesicular diseases and epidemic transient neonatal losses in swine. In addition, an asymptomatic SVA infection has also been reported in China, suggesting the existence of persistent SVA infection [30]. Currently, there is no corresponding commercial vaccine available for SVA, so it is necessary to design an effective vaccine for the prevention and control of SVA. In this study, we generated a His-tag-labeled SVA (rSVA-His) that expresses 6×His-tags stably on the viral capsid surface for the rapid purification of SVA vaccine antigens without negatively affecting viral phenotype. Specifically, the insertion of the 6×His-tag at the C-terminal of the SVA VP1 protein did not change the morphological structure of the virus. Additionally, rSVA-His showed a dynamic growth similar to that of its parental SVA. Neutralizing antibodies of picornaviruses mediate viral neutralization through binding to viral capsid proteins (VP1, VP2, and VP3) instead of viral functional proteins. The 6×His-tag expressed on the capsid of rSVA-His allowed us to rapidly purify the viral capsid protein by IMAC and to remove viral non-structural proteins and other cell-derived contaminants. SVA infection elicited an early and robust virus-neutralizing antibody response, which paralleled the reduction in viremia and the resolution of the disease [31]. More importantly, the neutralizing antibody titer induced in the rSVA-His immunized rabbits indicated that the insertion of the 6×His-tag does not affect the immunogenicity of SVA. However, further research will be needed to evaluate the protective capacity of purified rSVA-His in pigs. We think that rSVA-His can be used as a suitable immunogen to prevent SVA infection in pigs.

The reverse genetics system has been widely used in vaccine development and antiviral drug screening. In our previous studies, we successfully constructed an SVA recombination-deficient virus variant SVA-I212V-S460L, which has two nonsynonymous mutations in the RNA-dependent RNA polymerase (RdRp)-encoding region of the SVA genome. This mutant strain was proven to have high fidelity and can be considered as a suitable inactivated vaccine seed virus to ensure the stability of virus replication. We also identified that the G-H loop of FMDV effectively displays the protective neutralizing epitopes of other FMDV serotypes, suggesting that the C-terminus of SVA VP1 protein is permissive for foreign gene insertion [32]. Here, we introduced the 6×His-tag into the SVA-I212V-S460L infectious clone and demonstrated that the tag could be stably expressed on the viral capsid protein without affecting viral replication and immunogenicity. The recombinant tagged virus was purified by affinity chromatography in a Ni-NTA column to remove viral non-structural proteins, cell debris, host-cell-derived contaminants, and other impurities. The removal of non-structural protein could be advantageous to the application of the different infected from vaccinated animals (DIVA) test method.

Purification strategies of the virus include a variety of methods such as density-gradient ultracentrifugation, ultrafiltration, precipitation, two-phase extraction systems, and size exclusion chromatography. However, there are several drawbacks to the former methods, including being time-consuming and scale-restricted, with viral infectivity being lost. Chromatography-based virus purification strategies provide a convenient and practical choice for recovering viruses from cell culture [33]. For example, he 6×His-tagged FMDV bound to Ni^2+^ affinity columns resulted in one-step purification for developing novel marker vaccines [34]. This one-step purification strategy can also benefit the vaccine product development process as downstream processing contributes to the cost-effectiveness of an SVA vaccine.

The stability of exogenous gene insertion depends on the nature of the viral genome, the site of insertion and the size of the insert, and the viral recombination rate [35]. The insertion size of the exogenous genes for picornavirus is limited to inserts of about 300 bps or smaller, which are stable upon serial passages in tissue culture [36]. The size of the affinity tag is in the range that can accommodate the exogenous gene. Here, we used the recombinant virus SVA-I212V/S460L as the backbone of rSVA-His to solve the problem of exogenous gene removal in the viral genome. At present, there are more than a dozen tags commonly used for chromatographic purification. The 6×His-tag and Strep-tag were selected in our research to construct the recombinant tagged SVA. We also constructed rSVA-Strep with recombinant Strep-tag, which has similar characteristics to rSVA-His (data not shown). However, the price of 10 mg protein purified by Strep-Tactin-Sepharose is 10-fold higher than that by using Ni-NTA [37]. In this study, we performed an initial exploration of recombinant tagged virus purification. Some aspects needs to be optimized for the development of SVA inactivated vaccine.

## 5. Conclusions

In summary, we successfully constructed a His-tagged SVA mutant rSVA-His that expresses 6×His-tag stably on the surface of the SVA particle. Furthermore, we observed that the growth kinetics of rSVA-His are similar to that of its parental virus. The insertion of the 6×His-tag on the viral surface did not affect the immunogenicity of SVA. Importantly, the rSVA-His can be used for the rapid purification of SVA antigens for inactivated vaccines. These results provide a strategy for the development of a novel SVA vaccine.

## Figures and Tables

**Table 1:** Primers used for constructing pSVA-His.

	Primer	Sequence ^a^ (5′–3′)
1	In772U	CAC CAC CAC CAC CAC CAC AAG CAG AAG ATG CTG ATG CA
2	Ou750L	GTG GTG GTG GTG GTG GTG GTA GCT GCG AAA GGG AAG CAT G
3	SVA3241U	ACT CTG TCT CTT CCG TGC TTC
4	SVA5450L	CCC GGT CCG AGA CGT ACC ATC

^a^ Nucleotide sequence of the 6×His-tag is underlined.

## Data Availability

The data presented in this study are available on request from the corresponding author.
